# Dentists in Belgium

**Published:** 1869-01

**Authors:** 


Dentists in Belgium.—In the bill now before the House of Representatives it is proposed that dentists should possess the degree of Doctor of Medicine and Surgery. If the bill becomes law the dental profession in Belgium will be on a footing with the profession at large.

